# Epidemiological investigation of norovirus infections in Punjab, Pakistan, through the One Health approach

**DOI:** 10.3389/fpubh.2023.1065105

**Published:** 2023-03-14

**Authors:** Ammar Yasir, Yasir Mahmood, Muhammad Arsalan Yaqoob, Ubaid-ur-Rehman Zia, Claudia Munoz-Zanzi, Muhammad Masroor Alam, Muhammad Akib Warraich, Muhammad Hassan Mushtaq

**Affiliations:** ^1^Department of Epidemiology and Public Health, University of Veterinary and Animal Sciences, Lahore, Pakistan; ^2^Division of Environmental Health Sciences, School of Public Health, University of Minnesota, Minneapolis, MN, United States; ^3^Department of Virology, National Institute of Health, Islamabad, Pakistan; ^4^Department of Marketing, Rennes School of Business, Rennes, France

**Keywords:** diarrhea, acute gastroenteritis, One Health, noroviruses, genogroup II and III, Pakistan

## Abstract

**Introduction:**

Norovirus, mainly associated with acute gastroenteritis, is very contagious and can affect a vast range of species ranging from cattle, pigs, dogs, mice, cats, sheep, and lions to humans. It is a foodborne pathogen that mainly transmits through the fecal–oral route.

**Methods:**

This is the first-ever study conducted in Lahore and Sheikhupura districts of Punjab, Pakistan, to investigate noroviruses through the One Health approach. From January 2020 to September 2021, 200 fecal samples were collected from clinical cases of hospitalized patients and 200 fecal samples from sick animals at veterinary hospitals and local farms. In addition, 500 food and beverage samples were collected from street vendors and retail stores. A predesigned questionnaire was used to assess the risk factors and clinical characteristics of sick people and animals.

**Results and discussion:**

Overall, 14% of the human clinical samples were positive by RT-PCR for genogroup GII. All bovine samples were negative. Food and beverage samples were tested in pools, resulting in sugarcane juice samples positive for genogroup GII. Previous contact with acute gastroenteritis patients, sex, and presence of vomiting were found to be significant risk factors (*p* ≤ 0.05). The substantial number of diarrhea cases associated with noroviruses calls for additional studies to investigate the epidemiology and transmission and to improve surveillance.

## 1. Introduction

Noroviruses are single-stranded, non-enveloped, and non-segmented RNA viruses having icosahedral symmetry belonging to the family Caliciviridae. Based on the VP1 protein region, they are divided into 10 genogroups ranging from GI to GX (1). Noroviruses are the major cause of acute gastroenteritis among people of all ages but most commonly among children of <5 years of age (2). Globally, acute viral gastroenteritis is a major public health problem, and noroviruses cause an economic burden of $4.2 billion in direct health system outlays and $60.3 billion in societal costs per annum (3, 4). NoV is highly contagious, and a low dose of the virus is enough to cause infection, i.e., only 10–18 virus particles (5). Norovirus infects a wide range of hosts, including livestock animals, pets, marine mammals, rodents, and humans (6). Human infections are mostly caused by GI, GII, GIV, GVIII, and GIX, although GII is the most frequently detected genogroup in humans worldwide including Pakistan (7, 8), and the GIII genogroup is found in bovines (9). Animal noroviruses found in pigs, dogs, and cats are closely related to human strains and cluster in GII and GIV genogroups (10). Thus far, there is no evidence of zoonosis in noroviruses, although some serological evidence has been reported in some previous studies (6). Since the identification of noroviruses in animals and their relatedness to human strains, the question of crossing the species barrier has been raised. This was demonstrated by a study in which gnotobiotic pigs were infected with a human NoV strain, which raises the question of zoonosis (11). Applying the One Health approach for a multidisciplinary investigation is gaining insight to study the disease ecology of noroviruses (12). In humans, noroviruses can cause symptoms of severe vomiting, nausea, watery diarrhea, abdominal cramps, general malaise, and fever (13). The fecal–oral route is the main route of transmission, while other modes of transmission are person-to-person direct contact, consumption of contaminated food and water, exposure to contaminated environmental surfaces, and airborne vomit droplets. Factors such as the high NoV prevalence in the community, the shedding of infectious virus particles from asymptomatic individuals, and the high stability of the virus in the environment contributed to high levels of transmission (14). Bovine norovirus (BNoV) is classified into genogroup III (9), which is frequently detected in calf diarrhea cases and is a persistent threat to the livestock industry (15, 16). It causes mild diarrhea and transient anorexia in calves (17). Calf diarrhea is a substantial cause of economic losses in the livestock industry (18). The prevalence of bovine noroviruses ranges from 7.5 to 49.6% among different countries (19). Noroviruses are foodborne pathogens, and in Pakistan, foodborne illness investigations are focused traditionally on bacterial pathogens (20). Vegetables and fruits, as well as fast foods, baked products, and salads, present a potential health hazard due to unhygienic food handling practices (21–23). Previous studies conducted in Pakistan detected 16.1% and 19.5% prevalence for NoV in humans; although no studies have been conducted to investigate noroviruses in animals and foods and beverages (8, 24). Using the One Health principles to understand the ecology of noroviruses in Pakistan, the objectives of this study were to describe the presence of norovirus in people, animals, and food sources and the associated risk factors.

## 2. Materials and methods

### 2.1. Study design and sample collection

The study design was cross-sectional with a convenience sampling method. The study was approved by the Ethical Review Committee of the University of Veterinary and Animal Sciences, Lahore (059/IRC/BMR and DR/072). The study areas were the districts of Lahore and Sheikhupura in the Punjab Province. The study was conducted from January 2020 to September 2021.

Human stool samples were collected from children under 5 years of age from the Sheik Zaid and Children's hospitals of Lahore and two government Tehsil hospitals of Sheikhupura. Children included in the study were hospitalized for acute gastroenteritis as the primary illness and had watery diarrhea for fewer than 7 days with a frequency of three times or more per day along with dehydration (8). All the children were on oral or intravenous rehydration therapy. After informed written consent from the parents/guardians, up to 200 whole stool samples were collected from the diapers in a sterile container by applying a sterile plastic sheet and were stored at −80°C until further processing. A questionnaire was administered to the parents/guardians at the time of sample collection to collect demographic, clinical, and risk factors data.

Animal stool samples were collected from diarrheic calves presented for treatment at veterinary outdoor clinics and by visiting the local farms of Lahore and Sheikhupura which were conveniently accessible. The inclusion criteria were loose stool at the time of the visit and <1 year of age. Samples were collected from the rectum in a sterile container and were stored at −80°C until further processing. Enrollment and sampling continued until 200 stool samples were collected. Data collected included age, sex, fever status, duration and episodes of diarrhea, presence of quarantine area if any for disease animals, previous illness, and practice of visiting veterinary hospitals during the diarrhea illness.

Food and beverage samples were collected from different zones of Lahore district. Target food types including meat, vegetables, and fruits were collected from restaurants, local vendors, and retail shops. Sampling sites were selected based on convenience sampling by their location area and enrolled based on their willingness to provide food samples. Samples were collected using swabs following standard procedures for norovirus testing (25). Sampling continued until 500 samples of meat and 500 samples of other food items such as fast food, fresh juices, and salads were collected. For a given sample type, only one sample per vendor was collected. For meat, the swab was moistened in phosphate-buffered saline (PBS) and used for swabbing a defined area methodically in a horizontal, vertical, and diagonal direction. Whole juice samples and whole salads with a ratio of 1:10 for PBS were collected in a sterile container. All the food and beverage samples were tested in pools. Five samples of the same type were included in one pool.

#### 2.1.1. RNA Extraction and cDNA Synthesis

Fecal suspensions of each sample were prepared by diluting feces 1:10 (w/v) in ProSpecT™ sample diluent. These sample suspensions were mixed for 30 s and centrifuged at 8,000 rpm for 10 min at 4°C (8). Food and beverage samples were dipped and pressed in a sterile container having 9 ml of 1 × phosphate-buffered saline (PBS). RNA was extracted by the TRIzol method by TRIzol™ LS Reagent (Invitrogen^™).^ cDNA was synthesized at 42°C for 60 min and 70°C for 5 min with eluted RNA using the Thermo scientific™ RevertAid First Strand cDNA Synthesis Kit in a Thermal cycler (T100 Thermal Cycler, Bio-Rad, USA).

#### 2.1.2. Polymerase chain reaction (PCR)

Extracted DNA was amplified using Thermo scientific™ DreamTaq™ Green PCR Master Mix kit and specific primers set G2SKF CNTGGGAGGGCGATCGCAA and G2SKR CCRCCNGCATRHCCRTTRTACAT of product size 346 bp for genogroup GII (24) and for animals CBECu-F: AGTTAYTTTTCCTTYTAYGGBGA and CBECu-R: AGTGTCTCTGTCAGTCATCTTCAT primers set of product size 532 bp for GIII were used (16). For food and beverage samples, both primer sets of genogroup GII and GIII were used to identify the challenge virus. For the GII genogroup, PCR was optimized at 35 cycles using the following thermocycling conditions: 94°C for 30 s, 59°C for 45 s, and 72°C for 60 s followed by a final extension for 10 min at 72°C (24). For the GIII genogroup, PCR was optimized at 35 cycles using the following thermocycling conditions: 94°C for 3 min, 94°C for 30 s, 50°C for 30 s, and 72°C for 45 s followed by a final extension for 10 min at 72°C (16). PCR was carried out in a Thermal cycler (T100 Thermal Cycler, Bio-Rad, USA). PCR products were amplified at 2% agarose gel and were observed under UV light. All the samples were run with positive controls obtained from the National Institute of Health (NIH), Pakistan, and the Institute of Animal Husbandry and Veterinary Science, Henan Academy of Agriculture Science, China.

### 2.2. Statistical analysis

All the samples were classified as positive and negative based on the PCR results. For each sample type, results were first analyzed to describe the proportion positive overall and by the district. The statistical association between sample positivity and demographic characteristics, clinical signs, and risk factors was evaluated using the chi-square test and Fisher's exact test with a 95% confidence interval (CI). A *p*-value of ≤ 0.05 was considered statistically significant. The odds ratio (OR) was used to quantify the strength of the association between each risk factor and sample positivity. Episodes of diarrhea and vomiting and the duration of symptoms data collected in continuous numbers were classified for statistical analysis. All analyses were conducted using Statistical Package for Social Sciences (SPSS ver. 20.0).

### 2.3. Phylogenetic analysis

From the humans' positive samples, three random samples (GIIUVAS1, 2, and 3) were selected for the phylogenetic analysis of evolutionary analysis by the maximum likelihood method. PCR products were submitted for sequencing to a commercial company (Advanced Biosystems, Lahore). The accuracy of data was confirmed by bi-directional sequencing. Previously published sequences of norovirus genogroup GII and genogroup GIII from humans, animals, and water obtained from the National Center for Biotechnology Information (NCBI) were used as reference sequences. The study sequences were aligned and compared with reference sequences using ClustalW. Unique nucleotide sequences generated from this study were deposited in GenBank under accession numbers ON596245, ON596246, and ON596247.

## 3. Results

### 3.1. Humans

A detailed description of the results is provided in Table 1. Among 200 participants enrolled in the study, 61% were in the age group of >16 months, while 39% were in <33 months of the age group; 60% of the samples were collected from male participants, while 40% were from female participants. Laboratory analysis of stool samples yielded a prevalence of 14% (28/200) overall, 15% (15/100) in Lahore district, and 13% (13/100) in Sheikhupura. Norovirus positive proportion was 13.9% (17/122) in children <16 months of age and 14.1% (11/78) in the older ones with a *p*-value of 0.9. Norovirus positivity was significantly greater in female patients (19.8%, 16/81) than in male patients (10.1%, 12/119) (*p*-value = 0.05). The disease positivity was more, 15.5% (13/84), in children with 7–12 episodes of diarrhea than in children with 1–6 episodes, 12.9% (15/116), per day (*p*-value = 0.6). Vomiting was present in 16.7% (19/114) of participants, while in 10.5% (09/86) of samples, vomiting was absent. From the Sheikhupura district, vomiting in children was found significantly associated with the disease (*p*-value = 0.03) with a percentage of 21.4% (09/42). The percentage of positive and negative cases with the presence or absence of abdominal cramps was almost the same, i.e., 13.9% (17/122) in cases with abdominal cramps and 14.1% (11/78) in cases without abdominal cramps. Fever was not common in the positive cases, and most of the cases were positive for the noroviruses without fever, i.e., 19.1% (13/68) without fever and 11.4% (15/132) with fever were positive. Most of the cases, i.e., 16.7% (15/90), in the study were evaluated for a moderate level of dehydration by the physician. Disease positivity was more in children who had symptoms from the last 4–6 days, i.e., 15.3% (09/59), than in the 1–3 days, i.e., 13.5% (19/141). The percentage of positive cases was greater in children who ate any type of meat, 14.4% (24/166), compared with no meat, 11.8 (04/34), but the difference was not statistically significant (*p*-value = 0.6). Previous contact with acute gastroenteritis patients was found significantly associated with norovirus infection (*p*-value = 0.001).

**Table 1:** Association of demographic characteristics, clinical signs, and potential risk factors with PCR results in children from Sheikhupura and Lahore districts.

**Demographic characteristics**	**Lahore**			**Sheikhupura**			**Total**		
	**Proportion positive**	***P*-value**	**Odds ratio (95% CI)**	**Proportion positive**	***P*-value**	**Odds ratio (95% CI)**	**Proportion positive**	***P*-value**	**Odds ratio (95% CI)**
**Sex**									
Male	08/67 (11.9%)	0.2	0.5 (0.1–1.5)	04/52 (7.7%)	0.1	0.3 (0.1–1.2)	12/119 (10.1%)	0.05	0.4 (0.2–1.0)
Female	07/33 (21.2%)			09/48 (18.8%)			16/81 (19.8%)		
**Age (months)**									
≤ 16	10/61 (16.4%)	0.6	1.3 (0.4–4.2)	07/61 (11.5%)	0.5	0.7 (0.2–2.3)	17/122 (13.9%)	0.9	0.9 (0.4–2.2)
≥17	05/39 (12.8%)			06/39 (15.4%)			11/78 (14.1%)		
**Clinical signs**									
**Episodes of diarrhea**									
1–6	05/42 (11.9%)	0.4	0.6 (0.2–2.0)	10/74 (13.5%)	0.7	1.1 (0.3–4.7)	15/116 (12.9%)	0.6	0.8 (0.3–1.8)
7–12	10/58 (17.2%)			03/26 (11.5%)			13/84 (15.5%)		
**Vomiting**									
Yes	10/72 (13.9%)	0.6	0.7 (0.2–2.4)	09/42 (21.4%)	0.03	3.6 (1.0–12.9)	19/114 (16.7%)	0.2	1.7 (0.7–3.9)
No	05/28 (17.9%)			04/58 (6.9%)			09/86 (10.5%)		
**Abdominal cramps**									
Yes	11/67 (16.4%)	0.5	1.4 (0.4–4.8)	06/55 (10.9%)	0.4	0.6 (0.2–2.1)	17/122 (13.9%)	0.9	0.9 (0.4–2.3)
No	04/33 (12.1%)			07/45 (15.6%)			11/78 (14.1%)		
**Fever**									
Yes	08/73 (10.9%)	0.06	0.3 (0.1–1.09)	07/59 (11.9%)	0.6	0.7 (0.2–2.5)	15/132 (11.4%)	0.1	0.5 (0.2–1.2)
No	07/27 (25.9%)			06/41 (14.6%)			13/68 (19.1%)		
**Dehydration**									
Mild	05/31 (16.1%)	0.8	1.1 (0.3–3.6)	08/79 (10.1%)	0.09	0.3 (0.1–1.2)	13/110 (11.8%)	0.3	0.6 (0.3–1.4)
Moderate	10/69 (14.5%)			05/21 (23.8%)			15/90 (16.7%)		
**Duration of symptoms (days)**									
1–3	11/73 (15.1%)	0.9	1.02 (0.2–3.5)	08/68 (11.8%)	0.5	0.7 (0.2–2.4)	19/141 (13.5%)	0.7	0.8 (0.3–2.0)
4–6	04/27 (14.8%)			05/32 (15.6%)			09/59 (15.3%)		
**Risk factors**									
**Playing area**									
Home	14/85 (16.5%)	0.3	2.7 (0.3–22.7)	09/69 (13.1%)	0.9	1.0 (0.2–3.5)	23/154 (14.9%)	0.4	1.4 (0.5–4.0)
Outside	01/15 (6.7%)			04/31 (12.9%)			05/46 (10.9%)		
**Animal contact**									
Yes	02/18 (11.1%)	0.6	0.6 (0.1–3.2)	02/37 (5.4%)	0.08	0.2 (0.05–1.2)	04/55 (7.2%)	0.09	0.3 (0.1–1.1)
No	13/82 (15.9%)			11/63 (17.5%)			24/145 (16.5%)		
**Hands washing**									
Yes	14/94 (14.9%)	0.9	0.8 (0.09–8.06)	11/63 (17.5%)	0.08	3.7 (0.7–17.7)	25/157 (15.9%)	0.1	2.5 (0.7–8.8)
No	01/06 (16.7%)			02/37 (5.4%)			03/43 (6.9%)		
**Milk**									
Animal source	08/37 (21.6%)	0.1	2.2 (0.7–6.6)	04/42 (9.5%)	0.3	0.5 (0.1–2.0)	12/79 (15.2%)	0.6	1.1 (0.5–2.6)
Breast feeding	07/63 (11.1%)			09/58 (15.5%)			16/121 (13.2%)		
**Meat**									
Yes	11/74 (14.9%)	0.9	0.9 (0.2–3.3)	13/92 (14.1%)	0.2	0.8 (0.7–0.9)	24/166 (14.45%)	0.6	1.2 (0.4–3.9)
No	04/26 (15.3%)			00/08 (0%)			04/34 (11.8%)		
**Food source**									
Restaurant	06/39 (15.3%)	0.9	0.9 (0.3–2.9)	13/85 (15.3%)	0.1	1.1 (1.07–1.2)	19/124 (15.3%)	0.4	0.7 (0.3–1.7)
Home made	09/61 (14.8%)			00/15 (0%)			09/76 (11.8%)		
**Type of juice**									
Fresh	05/45 (11.1%)	0.3	0.5 (0.1–1.7)	07/45 (15.6%)	0.4	1.5 (0.4–4.8)	12/90 (13.3%)	0.8	0.9 (0.4–2.0)
Packed	10/55 (18.2%)			06/55 (10.9%)			16/110 (14.5%)		
**Washing of fruits and vegetables**									
Yes	14/89 (15.7%)	0.5	1.8 (0.215.7)	10/68 (14.7%)	0.4	1.6 (0.4–6.5)	24/157 (15.3%)	0.3	1.7 (0.5–5.3)
No	01/11 (9.1%)			03/32 (9.3%)			04/43 (9.3%)		
**Source of drinking water**									
Untreated	05/28 (17.9%)	0.6	1.3 (0.4–4.3)	09/79 (11.4%)	0.3	0.5 (0.1–1.9)	14/107 (13.1%)	0.6	0.8 (0.3–1.8)
Treated	10/72 (13.9%)			04/21 (19.1%)			14/93 (15.1%)		
**Hospital visit during illness**									
Yes	15/86 (17.4%)	0.09	0.8 (0.7–0.9)	12/97 (12.4%)	0.2	0.2 (0.02–3.3)	27/183 (14.8%)	0.3	2.7 (0.3–21.7)
No	00/14 (0%)			01/03 (33.3%)			01/17 (5.9%)		
**Previous contact with acute gastroenteritis patient**									
Yes	08/30 (26.7%)	0.03	3.2 (1.06–10.07)	08/31 (25.8%)	0.01	4.4 (1.3–15)	16/61 (26.2%)	0.001	3.7 (1.6–8.5)
No	07/70 (10%)			05/69 (7.2%)			12/13 (92.3%)		
**Previous illness**									
Yes	11/74 (14.9%)	0.9	0.9 (0.2–3.3)	06/43 (13.9%)	0.8	1.1 (0.3–3.7)	17/117 (14.5%)	0.7	1.1 (0.4–2.5)
No	04/26 (15.4%)			07/57 (12.3%)			11/83 (13.3%)		

### 3.2. Bovines

A total of 100 samples were collected from the eight clinics and 100 from five farms. Approximately 71% (142/200) of samples were collected from calves of <6 months of age. Sex distribution was 42% (84/200) male patients and 58% (116/200) female patients. At the time of sample collection, 83% of the calves had a fever, 49% were off the feed, and 67% had a history of diarrhea. The frequency of loose stools was 3–4 times/day for 81% and 6–8 times/day for the rest. The data obtained from calves enrolled from the farms showed that only 9% of owners responded “Yes,” that is, they visit the hospital during diarrhea. Only 10% of the owners reported having quarantine areas for sick animals. All 200 bovine fecal samples were negative for genogroup GIII by PCR.

### 3.3. Food and beverages

Meat samples collected included mutton (*n* = 150), beef (*n* = 150), and poultry (*n* = 200). Whole food items collected from fast food stores included paratha rolls (*n* = 3), shawarma (*n* = 15), pizza (*n* = 12), frozen nuggets (*n* = 50), and frozen chicken wings (*n* = 20). Fresh juices included sugarcane (*n* = 45), black currant (*n* = 27), strawberry (*n* = 18), watermelon (*n* = 40), mango (*n* = 36), lemon water (*n* = 27), black plum (*n* = 19), labun (*n* = 36), plum (*n* = 34), and tamarind water (*n* = 18). Vegetables from salads included onion (*n* = 28), tomato (*n* = 14), garlic (*n* = 11), cucumber (*n* = 21), and cabbage (*n* = 26). From the 200 food and beverage pools tested, two pools were PCR positive for NoV genogroup GII. All other pools were negative for both GII and GIII genogroups. The two positive pools consisted of sugarcane juice and each sugarcane juice pool contained samples from five different vendors. The positive pool results indicated that at least one individual sample represented in each of the two pools was positive. Due to budget constraints, the individual samples from the positive pools were not tested. Pool positivity calculated from all the samples was 1% (2/200) and for juice, it was 3.33% (2/60).

### 3.4. Phylogenetic analysis

**Figure 1:**
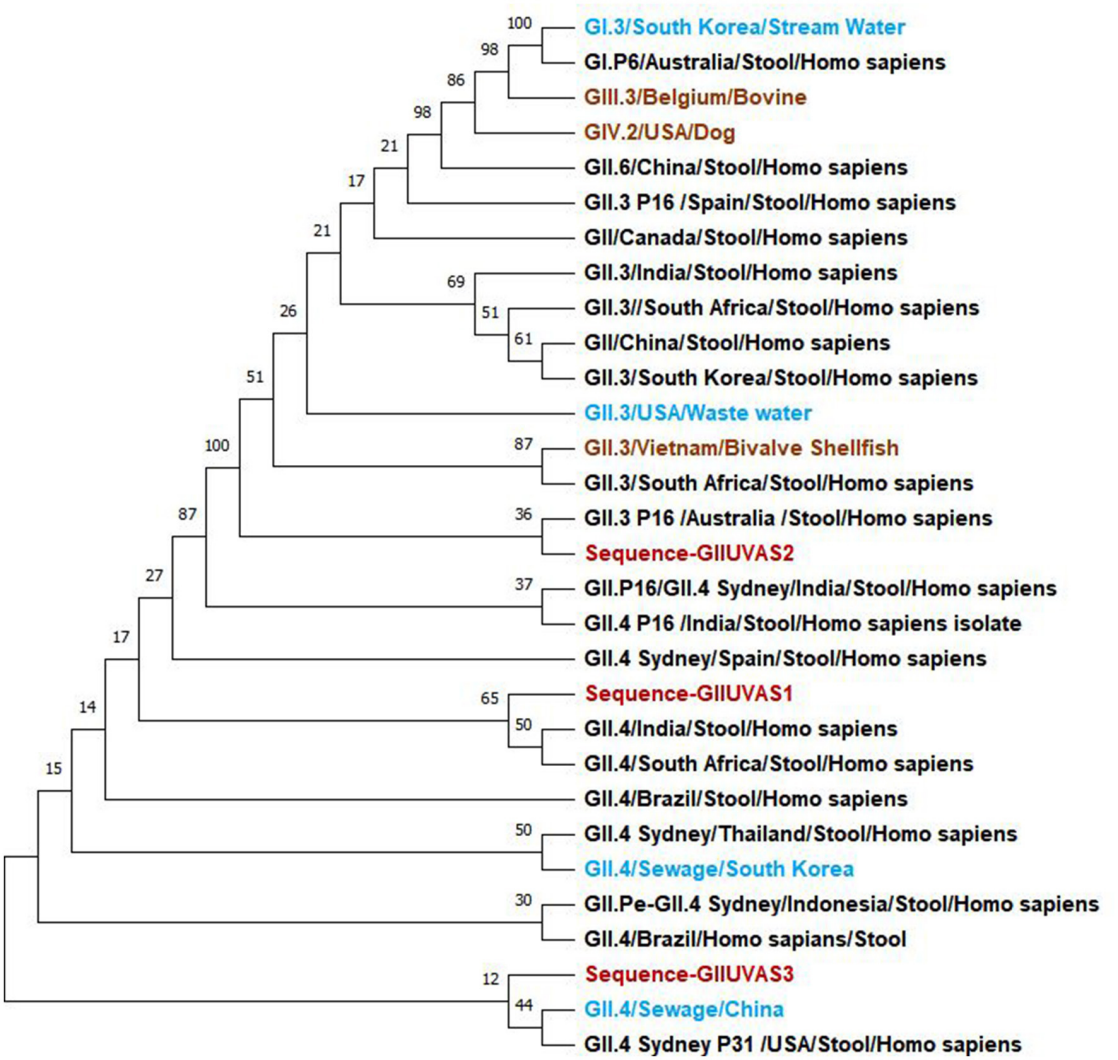
Phylogenetic relationships of norovirus GII genogroup isolates (red) from Pakistan compared to reference sequences in the NCBI database including the water (light blue), humans (black), and animals (brown) samples.

The evolutionary history was inferred using the maximum likelihood method and the Tamura-Nei model (26). The bootstrap consensus tree inferred from 100 replicates is taken to represent the evolutionary history of the taxa analyzed (27). Branches corresponding to partitions reproduced in <50% of bootstrap replicates collapsed. The percentage of replicate trees in which the associated taxa clustered together in the bootstrap test of 100 replicates is shown next to the branches (Figure 1) (27). Initial tree(s) for the heuristic search were obtained automatically by applying Neighbor-Join and BioNJ algorithms to a matrix of pairwise distances estimated using the Tamura-Nei model, and then selecting the topology with a superior log-likelihood value. This analysis involved 18 nucleotide sequences. There were a total of 306 positions in the final dataset. Evolutionary analysis was conducted in MEGA11 (28). Study samples of the genogroup GII strains were associated with the GII.3 and GII.4 of the reference sequences.

## 4. Discussion

The main objective of the study was to investigate the presence and ecology of noroviruses along with the identification of the potential risk factors using the One Health approach by collecting samples from humans, animals, food, and beverages. Previous studies conducted in Pakistan had only investigated the noroviruses in humans, and this was the first study exploring the noroviruses in animals and food and beverages. The genetic diversity of the circulating strains in Pakistan was also identified through phylogenetic analysis by partial sequencing of three samples from humans. The strains found in our study were associated with GII.3 and GII.4 when compared with the reference sequences from the National Center for Biotechnology Information (NCBI) by the maximum likelihood method. Overall, the prevalence in humans for genogroup GII was 14% varying at the district level but there was no positive sample detected from bovines. From a wide range of food and beverage samples investigated, samples from sugarcane juice, tested in pools, were found positive for genogroup II (GII) and negative for genogroup III (GIII).

In humans, previous studies have reported a prevalence of 9.9% in Karachi alone, and 16.1% and 19.5% in studies conducted in multiple cities (8, 24, 29). Prevalence data reported in this study falls in the range of other studies conducted in similar settings such as 10.3% in India (30), 5.1% in Bangladesh (31), 11.2% in Taiwan (32), and 10% in Yemen (33). Many of the previous studies tested the GI and GII genogroups but in this study, we have only tested samples for the GII genogroup which is the most prevalent genogroup in humans at the global level including Pakistan (8, 31, 34, 35). A study conducted in the neighboring country, India, reported 82.5% of cases of genogroup GII as compared to 12.5% of cases for the GI genogroup (30). In most of the studies reported here, the target population was under 5 years of age and was hospital-based. Our study confirms the important role of norovirus infections as the cause of acute pediatric gastroenteritis in Pakistan.

The difference in the prevalence of disease between our study and the previous studies might be because they have studied both genogroups GI and GII, and we only studied GII to demonstrate that the GII genogroup is the most prevalent in Pakistan associated with noroviruses as confirmed by the various studied mentioned earlier. Most of the previous studies were also aimed to study other viruses but our study was purely designed to study noroviruses. They used real-time PCR, while we used the conventional PCR which is less sensitive as compared to the aforementioned. Due to limited resources, broad objectives of the study, and previous reports of the high prevalence of the GII genogroup, the study was designed to obtain an idea of the circulating virus through the One Health approach.

Through the phylogenetic analysis, the noroviruses detected in this study were genetically more associated with genogroups GII.3 and GII.4. From the risk factors analysis, previous direct contact with the acute gastroenteritis patient was a significant risk factor for infection which has also been reported by other studies (36, 37). The odds ratio reported in the previous study was 14.23 [95% CI: 6.5–31.0], and in our study, it was 3.7 [95% CI: 1.6–8.5]. Norovirus is very contagious and when a person comes in contact with an infected person while caring for them and/or sharing food or utensils which may have been contaminated by feces or vomit particles, there is a high chance that the other person will become infected (36). According to Centers for Disease Control and Prevention (CDC), infected individuals shed billions of norovirus particles and one can contract the infection by a few particles which can also be transmitted through the air.

In our study, the positive samples were detected more in female participants than in male participants, whereas a previous study conducted in Pakistan reported more prevalence in male participants than in female participants, which might be due to the larger sample size of the male participants compared to the female participants (8). In this study, the ratio of vomiting and fever presence was less as compared to the other studies, which shows the asymptomatic importance of norovirus infections (8). The risk factors evaluated in this study were selected based on findings from previous studies (36, 38), in which they were found to be associated with infection. While our study did not identify many statistically significant risk factors, but it is crucial that factors such as poor hand hygiene and consumption of contaminated water play a critical role in the transmission of noroviruses, particularly in low-income countries (37, 39). In this study, results were also non-significant for breastfeeding; although, in literature, it has a protective role against norovirus infection due to the norovirus-specific immunoglobulin A antibody in breast milk (40).

In our human study, several limitations can also be noted including the small sample size and selected study participants (hospitalized children under 5 years of age) which should be broadened in further studies as children may have a limited range of exposures in terms of food and other risk factors included in this study. We were also not able to sequence many samples due to budget constraints which need to be broadened to better understand the genetic variability of the norovirus strains circulating in Pakistan.

To our knowledge, this was the first study in Pakistan to investigate noroviruses in animals. Neonatal calf diarrhea is one of the main causes of economic loss in the livestock industry (41). According to the Economic Survey of Pakistan 2021–2022, livestock is contributing ~14% to the national Gross Domestic Production (GDP) with more than 8 million rural families engaged in livestock production as their living source. Diarrhea is a frequent and growing concern leading to 24–63% of calf mortality in Pakistan (42). In Pakistan, the main etiological agents for calf diarrhea are rotavirus, coronavirus, enteropathogenic *E. coli*, Salmonella species, and Cryptosporidium, and most of the previous research is based on these agents (43, 44). Bovine norovirus has been proposed as one of the possible etiologies of calf diarrhea (45). They are also reported from the neighboring countries of Pakistan such as Iran and China (16, 46) which shows the threat of transboundary infection. Due to the ever-increasing trade in the livestock industry, now diseases are not limited to boundaries. The main objective of the study was to investigate the role of noroviruses in calf diarrhea as it is an emerging threat to the livestock industry worldwide. In future, further extensive investigation is recommended on a larger scale specifically during the calf diarrhea outbreaks to develop effective and realistic control and preventive strategies. From the 200 stool samples collected from the calves, there was no positive sample by RT-PCR. Many factors might be involved in the negative results which need to be addressed in future studies. First, no previous study was conducted on bovine noroviruses in Pakistan; hence, there might be a possibility that the virus may not be circulating despite reports of 20.4% prevalence in China and 39.5% in Iran which share borders with Pakistan (46, 47). Second, the sample size of our study was small (n=200), so there might be a possibility that the animals we screened were negative. This issue can be tackled by designing a nationwide study to assess the burden of noroviruses or can be combined with ongoing diarrheal illnesses' investigations.

Very little research has been conducted to explore the ecology of non-human populations. Close genetic relations between humans' and animals' noroviruses and the lack of information about the origin of new norovirus genotypes in humans have initiated the discussion about the possible routes for inter-species transmission (6). Up until now, there has been no evidence of the transmission of the virus from animals to humans; although, there are some serological hints observed in different studies for the possibility of transmission from animals to humans (48–50). In some studies, human noroviruses have been detected from animal stool samples (51, 52), and some experimental settings using animal models have also confirmed the ability to cross the species barrier (21, 53). To further investigate these findings, we adopted the One Health approach to better understand the relationship between norovirus ecology in humans and animals.

This study was also the first to investigate the presence of noroviruses in food and beverages in Pakistan. The sugarcane juice samples collected from local street food carts were found positive in our study. There can be multiple sources of contamination like the hands of the vendor and water or ice used. In our study, most of the food and beverage samples were negative, which is in accordance with food safety, but still, there is a risk of food contamination because the presence of noroviruses in humans is quite alarming and needs further investigation to assess the multiple risk factors associated with the disease. Surveillance, monitoring, and infection control system need to be strengthened for this purpose. However, some limitations can also be encountered in the food and beverages section of our study, such that we have analyzed samples in the pools, and the methods used for analysis are less sensitive.

Food and beverage products can be contaminated by fecal or vomitus material during the production process or by the hands of infected human beings (54). A study conducted by the Center for Food Safety, University of Georgia, to find out the transfer rate of norovirus from dry and wet stainless steel surfaces, found a transfer rate of more than 50% from wet surfaces as compared to dry ones. Depending on the type of water, NoV was able to persist in water for 60–728 days (55). Mostly in Pakistan, sugarcane juice machines are made of stainless steel and the surfaces remain wet all the time, so this might be a possible reason for the high persistent time for the virus to contaminate the sugarcane juice. Transmission from sugarcane juice can also be accompanied by multiple factors, including the contamination of ice (56), contaminated water usage for washing utensils, and using the same glass for many users (which is the most common practice in Pakistan) in which saliva can play an important role in the transmission of viruses (57). A similar study examining the presence of NoV in vegetables was performed by Cheong et al. However, only one spinach sample was found positive for NoV (58) which shows its low prevalence in vegetables which needs further investigation along with the large sample size enrollment and a better understanding of the diagnostic tests. Using conventional RT-PCR and manual extraction of viruses can be the other reasons for the no detection of noroviruses due to the low sensitivity of the processes.

## 5. Conclusion

This study concludes that noroviruses are contributing a significant part in childhood diarrhea cases with 14% positivity among the collected human diarrheal samples. All animal samples were negative. From the food and beverage samples, sugarcane juice samples were found positive with the recommendation to conduct further studies to roll out the associated risk factors with the noroviruses. This study provides an example of an interdisciplinary investigation of NoV at the human–animal–environment interface using the One Health principles; although little association was found in most of the risk factors, this study can be set as a pilot for the further investigation through the One Health approach which can provide much-needed insight into the prevalence and associated risk factors of norovirus infections.

## Data availability statement

The datasets presented in this study can be found in online repositories. The names of the repository/repositories and accession number(s) can be found in the article/supplementary material.

## Ethics statement

The studies involving human participants were reviewed and approved by Ethical Review Committee of the University of Veterinary and Animal Sciences, Lahore. Written informed consent to participate in this study was provided by the participants' legal guardian/next of kin. The animal study was reviewed and approved by Ethical Review Committee of the University of Veterinary and Animal Sciences, Lahore. Written informed consent was obtained from the owners for the participation of their animals in this study.

## Author contributions

Conceptualization, methodology, validation, formal analysis, investigation, resources, data curation, writing the original draft preparation, and project administration: AY and MH. Writing, reviewing, and editing: AY, YM, MY, U-u-RZ, CM-Z, MA, MW, and MH. Supervision: MH. All authors have read and agreed to the published version of the manuscript.
